# Supplementary material for: Chromothripsis during telomere crisis is independent of NHEJ, and consistent with a replicative origin
Source: Genome Res. 2019 May;29(5):737–49. doi: 10.1101/gr.240705.118 (PMC6499312; doi:10.1101/gr.240705.118)
Supplement: Supplemental Material [file supp_gr.240705.118_Supplemental_file_1.zip › contigs/annotated_contigs/DB73/contig.3.DB73_length_844_mean_cov_14.5734597156.docx]

**DB73_length_844_mean_cov_14.5734597156**

```
GGGCACTTTGCTAATCCATAGAAAACAGGAGATGGTTCTTTGGGGAAGGAAAACTCAACCATAAAAAGGATTGTGAAGAACAGAGTCAG
 >chr12:93362435-93362779 + E=5e-195
TACTTTTATTTATTTCCTAGTCTTGAGCATAAATGAGCTTCTAGGAAAGTGGAAGCACTCTGTGAAGAGACAACGCCCACCATCTTGCT

CCCAGGGTAGCTTCTGTGGATGGTGGAGAGCTGAGCTATAAAATAGCACAATGTGTTACCCATGGAAGGGCAGTCCTGGTTGAGGTTCT

TAATTGTGAATGTACAGATTCTGTTGAATGAATTGTGATAACCTCCAATTATCAGGAAAGAATCAATTAGGGAGAAC|CCCATCCAAAA

TTTAAGAA|CATCTTTTTAGAGTAGAGCCCTAGACAGCAATTGGGGATTAGTAGAGAGTCTCTAGGCCCCCAGGAAGGGGAATGCTAAG
         >chr12:93355191-93355417 - E=5e-121
ACTTCACAGGGCTCTAGTTGGGTAGAGCTGGGTTTTAGCTCTTGAGGCAGAAGTTCAATAGTTCAATTACATAGACTAGCCTCCCATAG

TGAGATTTAGGAGAGTATTATAACTCAGTGGATCCCATACAGGGATCACAGAT|ACTG|CCCCACCCCTATCTCCCTTTGCTGACTCTT
                                                      >chr12:93350695-93350954 - E=6e-1
TTTGGACTCAGCCCACTTGCATCCAAGTGAAATAAACAGCCTTGTTGCTCACACAAAGCCTGTTAGTGGACTCTCTTCACACAGAGGGG
44
CATGACATTGATATTCTGGAATGTTAAATATGTTACAAAGTAAATTCATGATGCCAGAGTTAAAAACAAAAACATGACGGGGCTTGGTG

GCTCACGCCTGTAATCCCAGCACTTTGGGAGGCCGAGGCAGGCAGAT
```
